# Cyclodipeptides: An Overview of Their Biosynthesis and Biological Activity

**DOI:** 10.3390/molecules22101796

**Published:** 2017-10-23

**Authors:** Awdhesh Kumar Mishra, Jaehyuk Choi, Seong-Jin Choi, Kwang-Hyun Baek

**Affiliations:** 1 Department of Biotechnology, Yeungnam University, Gyeongsan, Gyeongbuk 38541, Korea; awadhesh.biotech07@gmail.com (A.K.M.); stanza15@paran.com (J.C.); 2 Department of Biotechnology, Daegu Catholic University, Gyeongsan 38430, Korea; sjchoi@cu.ac.kr

**Keywords:** aa-tRNAsynthetase, cyclodipeptides, cyclodipeptide synthase, non-ribosomal peptide synthetase, tailoring enzyme

## Abstract

Cyclodipeptides (CDP) represent a diverse family of small, highly stable, cyclic peptides that are produced as secondary functional metabolites or side products of protein metabolism by bacteria, fungi, and animals. They are widespread in nature, and exhibit a broad variety of biological and pharmacological activities. CDP synthases (CDPSs) and non-ribosomal peptide synthetases (NRPSs) catalyze the biosynthesis of the CDP core structure, which is further modified by tailoring enzymes often associated with CDP biosynthetic gene clusters. In this review, we provide a comprehensive summary of CDP biosynthetic pathways and modifying enzymes. We also discuss the biological properties of some known CDPs and their possible applications in metabolic engineering.

## 1. Introduction

Natural peptide products are one of the most dynamic sources of medicinally significant compounds [1]. Cyclic dipeptides or cyclodipeptides (CDPs), also called 2,5-diketopiperazines, are the smallest cyclic peptides frequently found in nature, and are mainly synthesized by microorganisms [2]. CDPs are a class of cyclic organic compounds in which the two nitrogen atoms of a piperazine 6-membered ring form amide linkages. The mainframe structure of CDPs is a CDP scaffold generated by the condensation of two α-amino acids. The nomenclature of CDPs is indicated by the three letter code for each of the two amino acids, plus a prefix to designate the absolute configuration, e.g., cyclo(l-Xaa-l-Yaa). CDPs can be configured as both *cis* and *trans* isoforms, but *cis* configurations are predominant [3].Various amino acid modifications confer diversified chemical and biological functions. CDPs exhibit better biological activity than their linear counterparts due to their higher stability, protease resistance, and conformational rigidity, all factors that increase their ability to specifically interact with biological targets [4,5]. They constitute a large class of secondary metabolites produced by bacteria, fungi, plants, and animals [1,2,6,7]. The available data indicate that approximately 90% of CDP producers are bacterial [7]. CDPs and their derivatives exhibit a broad range of biological activities, such as bacterial quorum sensing, and antibacterial, antimicrobial, anticancer, and radical-scavenging properties. They have also been developed to carry biologically active molecules across the blood-brain barrier [1,8,9].

**Figure 1:**
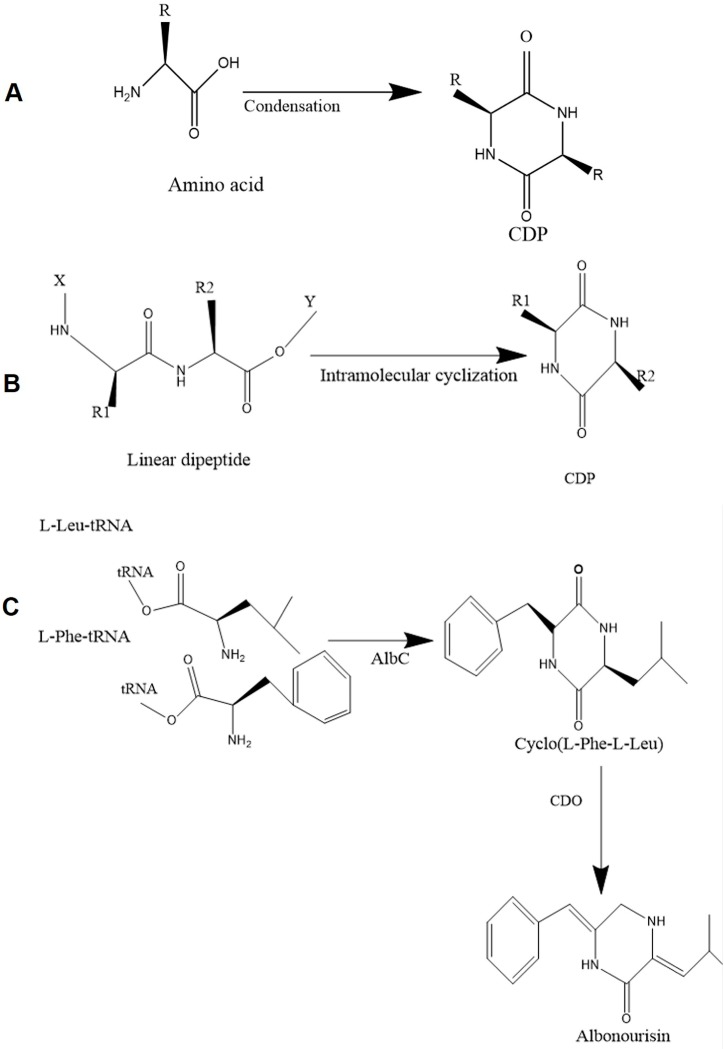
Structure and synthesis of the cyclodipeptide (CDP). CDP is synthesized chemically through (**A**) self-condensation of amino acids and (**B**) intramolecular cyclization of linear dipeptides. R, R1 and R2 represents a side chain, specific to each amino acid; (**C**) Biosynthesis of CDPs by CDP synthases (CDPSs) and subsequent modification by associated tailoring enzyme. Albonoursin [Cyclo(∆Phe-∆Leu)] (AlbC), is an antibacterial peptide produced by cyclo(l-Phe-l-Leu) and tailoring enzyme CDO (cyclicdipeptide oxidase). AlbC is a CDPS and Δ represents the dehydrated form of CDP.

The CDP scaffold can be synthesized either by purely chemically means using different solid-phases or under reflux conditions in solution [1,10] or more naturally, by biosynthetic enzymes called non-ribosomal peptide synthetases (NRPSs) and CDP synthases (CDPSs; [7,11]) (Figure 1). Common chemical synthesis of CDPs includes the condensation of individual amino acids at high temperature. Dipeptides substituted with an amine at one terminus and an ester at the other can also spontaneously cyclize to form a 2,5-DKP. However, conditions must be optimized carefully in order to force a cyclization reaction and to limit racemization. This is the procedure most commonly used for the chemical synthesis of CDP. Cyclization of amino dipeptide esters can also be carried out under thermal conditions, normally by refluxing them in high boiling solvents such as toluene or xylene for 24 h [1]. In addition, CDPs are often products of unwanted side reactions or degradation products of oligo- and polypeptides in processed food and beverages [2,12]. They are frequently formed during the chemical degradation of products in roasted coffee [13], stewed beef [14], and beer [15]. Non-enzymatic processes can also lead to the formation of functional CDPs in various organisms, for example, cyclo(l-His-l-Pro) is found throughout the central nervous systems of mammals [16]. This, cyclo(l-His-l-Pro) was the first active CDP and detected in human urine in 1965 [17]. In this review, we will highlight the CDP biosynthetic machinery and the associated modifying enzymes crucial for their biological activities.

## 2. Biological Mechanisms of CDP Formation

CDPs are commonly synthesized from amino acids by various organisms, including mammals, and are considered secondary functional metabolites or side products of terminal peptide cleavage. Several CDP biosynthetic pathways have been elucidated, and in general, they can be classified into non-enzymatic and enzymatic pathways.

### 2.1. Non-Enzymatic Pathways of CDP Formation

Cyclo(His-Pro) is an endogenous cyclic dipeptide that exists throughout the central nervous systems of various organisms, including mammals, and plays roles in a number of regulatory processes [16]. In mammals, cyclo(His-Pro) is derived from the precursor to thyrotropin-releasing hormone (TRH, pGlu-His-Pro). The TRH precursor, called TRH-Gly (pGlu-His-Pro-Gly), is first cleaved by pyroglutamate aminopeptidase, producing His-Pro-Gly, which is then non-enzymatically cyclized to cyclo(His-Pro). The proline induces constraints that promote the *cis*-conformation of the peptide bond between the histidine and the proline, thereby facilitating cyclization which generates the CDP scaffold. This mammalian CDP imparts the cytoprotective effect during NF-κB (nuclear factor kappa-light-chain-enhancer of activated B cells) and Nrf2 (nuclear factor like 2) signaling [16].

### 2.2. Enzymatic Pathways of CDP Formation

CDPs are commonly considered to be secondary metabolites. Some protease enzymes, such as dipeptidyl peptidases, cleave the terminal ends of proteins into generate dipeptides, which can naturally cyclize to form CDPs. Two unrelated biosynthetic enzyme families catalyze the formation of CDPs: NRPSs and CDPSs.

#### 2.2.1. NRPS-Mediated CDP Biosynthesis

CDPscaffolds can be synthesized by one or more specialized NRPSs, either through dedicated biosynthetic pathways or through the premature release of dipeptidyl intermediates during the chain elongation process. The NRPS genes for a certain peptide are usually organized in one operon in prokaryotes, and in a gene cluster in eukaryotes [18]. NRPSs are large modular enzymes, which simultaneously act as a template and as biosynthetic machinery. Each module is responsible for the incorporation of one amino acid into the final peptide, and can be further subdivided into the catalytic domains responsible for specific synthetic steps during peptide synthesis [19]. In each module, NRPSs consist of three necessary domains: an adenylation (A) domain; a thiolation (T) domain post-translationally modified with a 4′-phosphopantetheinyl (4′-Ppant) arm, also termed the peptidyl carrier protein (PCP) domain; and a condensation (C) domain, separated by short spacer regions of approximately 15 amino acids. The A domain selects, activates, and loads the monomer onto the PCP domain. Here, the thiol group of the 4′-Ppantarm of the T domain mediates the nucleophilic attack of the adenylated amino acid. Subsequent peptide bond formation between two adjacent T-bound aminoacyl intermediates is catalyzed by the C domains [11]. Another essential NRPS catalytic unit is the thioesterase (TE) domain, which is located in the C-terminus and catalyzes peptide release by either hydrolysis or macrocyclization. In addition, modification domains can be integrated into NRPS modules at different locations to modify the incorporated amino acids. Epimerization and N-methyltransferase domains are examples, which catalyze the generation of D- and methylated amino acids, respectively [20,21]. NRPSs rely not only on the 20 canonical amino acids, but also use several different building blocks, including non-proteinogenic amino acids, and this contributes to the structural diversity of non-ribosomal peptides and their differential biological activities [20]. CDPs synthesized by NRPSs can be further modified by tailoring enzymes, usually encoded by genes clustered with the NRPS genes. The majority of known NRPS-derived CDPs are produced by fungi, whereas few bacteria are recognized as NRPS-derived CDP producers.

Many CDPs can be formed by dedicated NRPS pathways, such as brevianamide F [22], erythrochelin [23], ergotamine [24], roquefortine C [25], acetylaszonalenin [26], thaxtomin A [27], gliotoxin [28], and sirodesmin PL [29]. In a few cases, CDPs can be formed by NRPSs during the synthesis of longer peptides, as truncated side products, as in the biosynthesis of cyclo(d-Phe-l-Pro) and cyclomarazine A [30,31].

#### 2.2.2. CDPS-Mediated CDP Biosynthesis

CDPSs are a new family of tRNA-dependent peptide bond-forming enzymes that do not require amino acid charging. CDPSs share a common architecture reminiscent of the catalytic domain of class-Ic amino acid tRNAsynthetases (aaRSs), for example TyrRS and TrpRS [32]. Both CDPSs and class-IcaaRSs comprise well conserved Rossmannfold domains along with a helical connective polypeptide 1 (CP1) subdomain. However, Class-IcaaRSs possess signature motifs involved in ATP binding (HIGH and KMSKS sequences) that are not present in CDPSs. In addition, CDPSs do not possess a distinct tRNA-binding domain, but rather contain a large patch of positively charged residues located in helix α4, which are important for the binding of aminoacyl-tRNA substrates. All these observed differences between CDPSs and their ancestral aaRSs result in unique enzymes for CDP biosynthesis.

**Figure 2:**
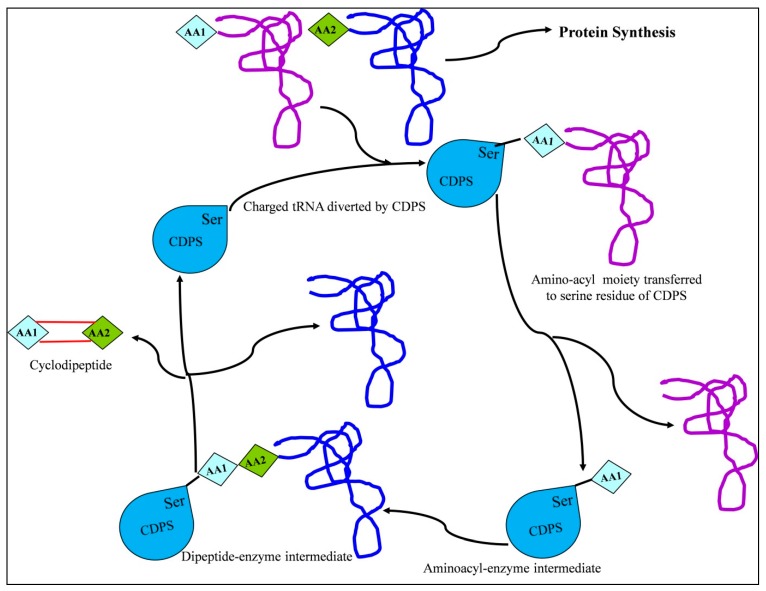
Biosynthesis of the CDP scaffold by the CDPS-dependent pathway (modified from Belin et al. [11]). Here, AA represents the amino acid.

CDPSs use amino acid tRNAs as substrates to catalyze the formation of CDP peptide bonds [11,33,34,35], diverting two aminoacyl-tRNAs from their essential role in ribosomal protein synthesis for use as substrates and catalyzing the formation of the two peptide bonds required for CDP formation [36]. The synthesis process is initiated by the binding of the first aminoacyl substrate, likely involving ionic interactions between the negatively-charged ribose–phosphate tRNA backbone and the positive charges in helix α4 [32,37]. Hence, by using aminoacyl-tRNAs as substrates, CDPSs represent a direct link between primary and secondary metabolism. The catalytic mechanism of CDPSs can be described using a ping–pong model (Figure 2). All CDPSs possess two surface-accessible pockets that contain active site residues important for substrate selection and catalysis. The different aminoacyl binding sites for the two aa-tRNA substrates are termed pocket 1 (P1) and pocket 2 (P2). Upon specific recognition of the first substrate, the first aminoacyl group is transferred to the conserved serine residue of P1. Here, interaction between the tRNA moiety and basic residues in the α4 helix generates an aminoacyl–enzyme intermediate [34,38]. In the meantime, the aminoacyl moiety of the second aa-tRNA interacts with P2 through the α6–α7 loop. Ultimately, the aminoacyl–enzyme intermediate reacts with the second aa-tRNA to generate a dipeptidyl–enzyme intermediate, which undergoes intramolecular cyclization through the involvement of a conserved tyrosine, leading to the CDP scaffold as the final product. These CDPs can also be modified by closely associated tailoring enzymes.

There are approximately 163 putative CDPS genes identified so far, and of these, 150 are reported in bacteria, distributed among six phyla (Actinobacteria, Bacteroidetes, Chlamydiae, Cyanobacteria, Firmicutes, and Proteobacteria). Most known CDPSs are found in Actinobacteria, with 77 CDPSs reported to date. Twelve CDPSs were distributed among four eukaryotic phyla (Ascomycota, Annelida, Ciliophora, and Cnidaria), and one archaeon (*Haloterrigena hispanica*) CDPS has also been reported [7,11,39]. Some bacterial CDPSs have been fully characterized, such as albonoursin in *Streptomyces noursei*, pulcherrimin in *Bacillus subtilis*, and mycocyclosin in *Mycobacterium tuberculosis* [11,40].

## 3. Comparison of CDPS- and NRPS-Dependent Pathways

Both the CDPS and NRPS systems are used to synthesize CDP metabolites in nature. CDPSs are small enzymes (~26 kDa), while NRPSs are large modular enzymes (>100 kDa) [11]. This size difference probably reflects the different strategies used to activate the amino acid carboxyl group required for peptide bond formation: NRPSs use A and PCP domains to recognize and activate amino acids in the form of PCP-bound aminoacyl thioesters, whereas CDPSs hijack aminoacyl-tRNAs, thereby eliminating the need to activate amino acids. The substrates of CDPSs are therefore limited to the 20 l-amino acids charged on tRNAs, whereas the range of amino acids that can be incorporated by NRPSs is much wider, including non-proteinogenic amino acids such as anthranilic acid in the synthesis of acetylaszonalenin, and 4-nitrotryptophan in thaxtomin biosynthesis [26,41]. Moreover, NRPS substrates can be altered on the enzyme by accessory domains, which introduce chemical modifications such as methylation (methylation domains, thaxtominsynthetase) or configuration changes (epimerization domains, erythrochelin synthetase), while in CDPS pathways, chemical modifications can only be introduced after CDP formation. Hence, wider structural complexity is found in CDPs synthesized via NRPS pathways (e.g., roquefortine, siderosmin, ergotamine) than in those synthesized via CDPS pathways. Moreover, NRPS-dependent pathways are prevalent in bacteria and fungi (but have not yet been identified in plants or animals), while CDPS-dependent pathways have been identified in bacteria (*Bacillus* sp., *Pseudomonas* sp.), fungi (*Gibberella zeae*, *Fusarium oxysporum*), protozoa (*Ichthyophthirius multifiliis*), and animals (*Nematostella vectensis*, *Platynereis dumerilii*).

## 4. CDP-Tailoring Enzymes and Their Functions

Tailoring enzymes that specifically modify CDP-containing natural products are usually associated with biosynthetic enzymes. Putative tailoring enzymes that modify the initially assembled CDPscaffold can be found in almost all NRPS and CDPS gene clusters, and are responsible for installing functional groups crucial for the biological activities of CDPs. In CDPS-dependent pathways, a large variety of different modification enzymes are found in close association with the respective CDPS genes [7,11], including different types of oxidoreductases, hydrolases, transferases, and ligases. The most prevalent putative tailoring enzymes in CDPS clusters are cyclic dipeptide oxidases (CDOs). CDOs are composed of two distinct small subunits that assemble into an apparent mega-dalton protein complex. Depending on the substrate, the CDO can sequentially perform one or two dehydrogenation reactions. The precise reaction mechanism for this has not been elucidated, although three different scenarios have been proposed: direct dehydrogenation, α-hydroxylation followed by loss of water, and imine formation with subsequent rearrangement of the enamine [42]. Known CDOs include at least seven distinct P450 enzymes, five different types of α-ketoglutarate/Fe^II^-dependent oxygenases, and three distinct flavin-containing mono-oxygenases.

In addition to oxidoreductases, a large number of different C-, N-, and *O*-methyltransferases, α/β-hydrolases, peptide ligases, and acyl-CoA transferases have been found in CDPS gene clusters in which different transcription factors belonging to the LuxR and MarR families, among others, are observed. They are usually involved in regulating various processes in response to environmental stimuli like toxic chemicals and antibiotics, which may hint at the biological functions of CDPS-dependent modified CDPs [43]. Regarding NRPS-dependent pathways, a similar variety of modification enzymes has been reported, and again, enzymes that modulate the oxidation of the CDP scaffold and side chains are the most numerous [11]. One distinguishing feature of fungal NRPS gene clusters is the prevalence of different prenyltransferases, which perform prenylations and reverse prenylations at various positions of tryptophan-containing CDP scaffolds [44]. Judging by the diverse set of putative modification enzymes found within NRPS and CDPS gene clusters (Table 1), it is assumed that highly modified CDPs represent a diverse family of microbial natural products with varied functions.

Both chemical synthesis and enzyme-catalyzed assembly are valid ways of providing suitable substrates for CDP tailoring enzymes. When using chemically synthesized substrates, CDP modification enzymes can be employed in chemoenzymatic and cell-free in vitro settings, as well as in feeding experiments, while whole-cell in vivo biosynthesis based on in situ substrate generation by NRPS or CDPS enzymes represents an alternative approach to obtain modified CDPs [52].

## 5. Rational Design of CDPs for Structurally Diverse Peptides

There are two common methods for rationally altering the structure of natural peptides currently in practice. First, the peptide backbone itself can be altered by changing the identity, number, or connectivity of the constitutive amino acids. Second, tailoring enzymes can be introduced to catalyze specific chemical modifications into already assembled peptide scaffolds, leading to the synthesis of functionally altered peptides [34].

DPs are normally quite limited with regards to peptide backbone modification, as they are composed of only two amino acid residues arranged with a predefined connectivity. Hence, the only possible diversification method is the alteration of monomer identity. Moreover, CDPS-derived CDPs have an additional limitation, because they use charged tRNAs as substrates, which means that they only contain 20–22 proteinogenic amino acids. CDPS specificity mainly depends on the identity of the aminoacyl moiety bound to the tRNA. Hence, diversification of the CDP scaffold can be achieved by changing either the building block carried by a certain tRNA or the sequence of a tRNA that is specific for a particular amino acid. In vitro transcription or standard mutagenesis techniques can be employed to introduce small specific sequence changes into tRNAs that do not affect their overall structure or aminoacylation [53].

Additionally, altering the proteinogenic amino acids loaded onto specific tRNAs to produce non-proteinogenic amino acids could be used to produce CDPs containing non-standard monomers. For this, a residue-specific incorporation strategy can be employed by omitting the natural amino acid of choice in the growth medium while providing a non-canonical analog. By combining this with the use of auxotrophs as expression hosts, it is possible to obtain high-level replacement [54]. Another strategy to alter the structure of natural peptide products is the tailoring of an already assembled peptide scaffold by specific modification enzymes [34]. This approach can be employed in CDPs derived from both pathways, and increases the feasibility of constructing artificial hybrid pathways for the production of highly modified CDPs.

## 6. Biological Activities

Due to their rigid cyclic structure, CDPs, unlike their linear counterparts, can withstand enzymatic degradation, and this has further prompted the development of CDP-based systems for biomedical applications [4]. In medicinal chemistry, CDPs are considered to be promising scaffolds for the design of a variety of active compounds for diverse biological functions because of the versatility associated with CDP functionalization [1]. These compounds display diverse and noteworthy biological effects, such as antibacterial [55], antitumor [56], antifungal [57], and antiviral [58] activity. For instance, cyclo(l-Phe-l-Pro) and cyclo(l-Phe-trans-4-OH-l-Pro) are antifungal compounds [59]. Erythrochelin, coprogen, and dimerumic acid are siderophores [23,60,61], while roquefortine C and acetylaszonalenin are mycotoxins [25,26]. Bicyclomycin and albonoursin are antibacterial agents [62,63], and thaxtomin A acts as a phytotoxin [27,64]. Brevianamide S exhibits selective antibacterial activity against *Mycobacterium bovis* bacillus Calmette-Guérin, suggestive of antitubercular potential [65]. Ambewelamides A and B, phenylahistin, and verticillin A exhibit antitumor properties [66,67]. Gliotoxin, a type of mycotoxin, and sirodesmin PL, a type of phytotoxin, have antibacterial, antiviral, and immune suppressive properties [68,69]. In addition, gliotoxin is also a potent inducer of apoptotic and necrotic cell death [70,71].

Some CDPs act as diffusible molecules involved in cell-to-cell communication, and may constitute a new class of quorum-sensing signals [72,73] or interspecies signals [74] in bacteria. Moreover, the CDP-based compound plasminogen activator inhibitor-1 (PAI-1) acts as a main serine protease inhibitor in humans [75,76], and the above mentioned cyclo(His-Pro), a metabolite of TRH precursor is directly involved in the control of high blood glucose levels [77]. Other CDP derivatives play important roles in cardiovascular and blood-clotting functions [77].

Further, CDPs have bioactive effects on their plant or animal hosts, and a role in trans-kingdom signalling has also been proposed. Three CDPs, cyclo(l-Pro-l-Val), cyclo(l-Pro-l-Phe), and cyclo(l-Pro-l-Tyr), are involved in the quorum-sensing-mediated promotion of plant growth by *Pseudomonas aeruginosa* [78]. Additionally, Chen et al. [79] have recently shown that cyclic dipeptides produced by the fungus *Eupenicillium brefeldianum* HMP-F96 induce extracellular alkalinization and H_2_O_2_ production in tobacco cell suspensions. Moreover, Wu et al. [80] reported that two CDPs, named cyclo(l-Pro-l-Pro) and cyclo(d-Pro-d-Pro), induced systemic disease resistance in *Nicotiana benthamiana* against *Phytophthora nicotianae* infections by activating a salicylic acid-dependent defence pathway.

## 7. Concluding Remarks

In conclusion, CDPs are usually biosynthesized from amino acids by tRNA-dependent cyclodipeptide synthases or non-ribosomal peptide synthetases. These represent complex mechanisms for secondary metabolism production. CDPs have attracted immense attention because they possess unique chemical and diverse biological properties such as antitumor, antifungal, antibacterial, and radical-scavenging activities. Further, CDPs are characterized by their resistance to proteolysis and improved interactions with biological targets, prompting the use of CDP-modified compounds in many pharmaceutical ingredients. In vivo or in vitro, a combination of biological and chemical pathways can generate specific hybrid target compounds, and this can enhance the natural diversity of CDPs. Although much is known about CDPs, many questions remain unanswered. For what purpose, and under which conditions, are specific modified CDPs produced in nature? Functional diversity is imparted by tailoring enzymes, but what kinds of modifications are introduced in which situations? In addition, we expect more CDPs will be identified in the near future, and could be employed in diverse biological applications.

## Figures and Tables

**Table 1:** Overview of different tailoring enzymes associated with gene clusters of the non-ribosomal peptide synthetase (1–7) and cyclodipeptide synthase (8–13) pathways, their putative functions, and the substrate CDPscaffold used by those enzymes.

Serial Number	Biosynthetic Pathway	Tailoring Enzymes	Putative Function	CDP Substrate	Reference
1	Thaxtomin A (*Streptomyces scabies*)	Cytochrome P450TxtC	Hydroxylation	c(WF)	[27,45]
2	Brevianamide (*Aspergillus fumigatus*)	Afu8g00240 Afu8g00230 Afu8g00220 Afu8g00200 Afu8g00190	Oxidative cyclization Oxidative cyclization Hydroxylation *O*-methylation Hydroxylation	c(WP)	[22]
3	Ergotamine (*Claviceps purpurae*)	CpP4501 CpCAT2 CpOX3	Hydroxylation Hydro peroxidation Oxidative cyclization	c(FP)	[24]
4	Meleagrin (*Penicillium chrysogenum*)	Pc21g15430 Pc21g15440 Pc21g15450 Pc21g15460 Pc21g15470	C3-reverse-prenylation *O*-methylation Oxidative cyclization *N*-hydroxylation α,β-dehydrogenation	c(WH)	[25]
5	Acetylazonalenin (*Neosartorya fischeri*)	AnaPT AnaAT	*C3*-reverse-prenylation *N*-acetylation	c(WF)	[26]
6	Gliotoxin (*Aspergillus* spp.)	GliC GliF GliG GliI GliM GliN	Oxidation Oxidation Sulfurization Cyclopropane-formation *O*-methylation *N*-methylation	c(FS)	[46]
7	CDP-alkaloids biosynthetic pathway (Fumitremorgin biosynthesis) (Aspergillus and Penicillium)	Prenyltransferases	Prenylation	c(WW)	[44]
8	Ditryptophenaline (*Aspergillus fumigatus)*	Cytochrome P450 DtpC	Dimerization	c(WF)	[47]
9	Nocazine pathway (Dehydrophenylahistin) (*Nocardipsis* spp.)	Cytochrome P450	α,β-dehydrogenation	c(ΔLF)/c(LΔF)	[48]
10	Albonoursin (*Streptomyces noursei)*	Cytochrome P450	α,β-dehydrogenation	c(FL)	[49]
11	Pulcherriminic acid (*Bacillus subtilis)*	Cytochrome P450Cyp134A1	Fe-dependent Oxidation	c(LL)	[37,50]
12	Mycocyclosin (*Mycobacterium tuberculosis*)	Cytochrome P450Cyp121	Dimerization	c(YY)	[33,42]
13	Neihumicin (*Nocardiopsis* spp.)	Ndas-1145	S-adenosyl-methionoine dependent *O*-methyltransferase	c(FF)	[51]

Substrate amino acids are represented by single letters; Δ represents the dehydrated form of CDP.
